# Board of Health Statistics Show Favor to Lady Bicyclers

**Published:** 1897-06

**Authors:** 


Board of Health statistics have begun to show favor to lady bicyclers. The Secretary of the Massachusetts Board gives figures that show, *pari passu* with the development of the so-called bicycle craze among women has come an equally steady decrease in the number of deaths among them from phthisis. We hope that the boards of health of other States will look into this matter and see if the results are the same everywhere. It is quite likely that the taking of more out-door exercise by women has led to this good result.

